# Benefits of Complementary Therapies During Pregnancy, Childbirth and Postpartum Period: A Systematic Review

**DOI:** 10.3390/healthcare12232481

**Published:** 2024-12-09

**Authors:** Consolación Lima-De-La-Iglesia, Eleonora Magni, Alicia Botello-Hermosa, María Dolores Guerra-Martín

**Affiliations:** 1 Obstetrics and Gynecology Service, Virgen Macarena University Hospital, 41009 Seville, Spain; conlimde@alum.us.es; 2 Faculty of Nursing, Physiotherapy and Podiatry, University of Seville, 41009 Seville, Spain; guema@us.es; 3 Institute of Biomedicine of Seville (IBiS), 41013 Seville, Spain; 4 Research Group CTS969: Care Innovation and Health Determinants, 41009 Seville, Spain; 5 Research Group SEJ066: Women, Wellbeing and Citizens, 41009 Seville, Spain

**Keywords:** pregnancy, delivery, postpartum period, complementary therapies, systematic review

## Abstract

Background/Objectives: The current trend in maternal health is to foster more natural and less medicalized therapies, with increasing interest in complementary therapies. This study has analyzed the benefits of complementary therapies during pregnancy, delivery and the postpartum period. Methods: A paired systematic review was carried out (PROSPERO: CRD42024543981). The following databases were consulted: PubMed, Scopus, Web of Science and CINAHL. Inclusion criteria were randomized clinical trials about complementary therapies in women during pregnancy, delivery and/or the postpartum period. The risk of bias of the clinical trials was evaluated using the revised Cochrane Risk of Bias tool for randomized trials version 2 (RoB-2). Results: A total of 1684 studies were found, with a final selection of 17. The most studied symptom was pain. Hydrotherapy, perinatal Swiss ball exercises, acupressure, virtual reality and foot reflexology provided a significant reduction in pain during labor. Similarly, aromatherapy, electroacupuncture and massage have shown significant benefits in pain management after Cesarean. Yoga, when practiced during pregnancy, effectively reduces anxiety and depression, with similar benefits observed when practiced during the postpartum period. Conclusions: Complementary therapies improve pain, anxiety and depression management across maternal health phases.

## 1. Introduction

Women’s health during pregnancy, delivery and the postpartum period represents a priority for the World Health Organization (WHO) [1]. The normal physiological changes experienced by women can cause common symptoms and problems, along with possible more serious medical complications [2] that negatively affect their quality of life [3]. Among the most common problems during pregnancy, we can mention nausea and vomiting [4], musculoskeletal disorders [5,6] and psychological discomforts such as anxiety, stress and depression [7].

On the other hand, and due to its intrinsic characteristics, pregnancy is associated with pain, either due to vaginal delivery [8] or to postoperative pain after a Cesarean section (C-section). There can also be anxiety and stress [9], which can contribute to a negative delivery experience [10], to increased epidural analgesia use [11], to influencing newborns’ well-being [12] and to increasing the risk of developing postpartum depression [13]. With 14% prevalence, the most common complication during the postpartum period is postpartum depression [14], which is associated both with the delivery experience (that is to say, anxiety and stress) [13] and with the psychological symptoms experienced during pregnancy [7,14].

In recent decades, complementary therapies (CTs) have been integrated into traditional medicine (that is to say, pharmacological interventions) with the purpose of contributing to maintaining maternal health [15,16]. CTs are defined as a broad set of health practices not included in a country’s own tradition and not integrated into the predominant health system, but which are used along with conventional medicine to improve people’s health and well-being [17], providing holistic care [18,19].

Acupressure and acupuncture are two of the CTs most resorted to during pregnancy; their use to alleviate nausea and vomiting has proved to be more effective than the conventional approaches [20]. These two therapies have also been employed as tools to mitigate pain, showing effectiveness in reducing it during delivery [21,22]; however, no clear evidence has yet been found as to their efficacy for postpartum pain management [23]. Aromatherapy is also among the CTs most used for pain; it has proved to alleviate both pain during delivery [24] and anxiety [25]; nevertheless, no conclusive evidence has been found about its effectiveness during the postpartum period [23].

In a review about the effectiveness of CTs for pain management after C-sections [16] it was found that, although without reaching any solid level of evidence, acupressure and acupuncture combined with analgesics can reduce pain 12 and 24 h after a C-section, as is the case with aromatherapy alone. Other CTs such as relaxation, electromagnetic therapy and Transcutaneous Electrical Nerve Stimulation (TENS) were identified in that same review; they also seem to exert positive effects in this phase, reducing pain 24 h, 12 and 24 h and even one hour after a C-section, respectively.

Smith et al. (2022) have also studied pain management with CTs during the postpartum period (associated with episiotomy, low back pain or pain after a C-section); they found that, among the CTs investigated, massage, cupping and herbal tablets and ointments were the ones that seemed to exert a positive effect on pain [23].

Smith et al. (2018) researched the CTs that might be beneficial for reducing pain during delivery and found positive effects (although with very low evidence quality) in favor of massage, hot compresses and thermal manual methods [26].

Other types of CTs that have been used to alleviate psychological symptoms such as stress and anxiety include yoga [27] and music-based interventions [28]. In a recent review about the effectiveness of yoga during pregnancy [27], it was found that yoga-based interventions not only reduced anxiety, depression and perceived stress but also exerted a positive influence on delivery duration and increased the probability of vaginal delivery. Hunter et al. (2023) also studied anxiety; they found that music-based interventions might be an efficient tool to reduce both anxiety and pain during delivery, proving to be more effective if used with ear pods, if combined with other therapies (such as massage or acupressure) and if applied during a C-section [28].

In general, most of the authors of the aforementioned studies [16,23,28] agree on the fact that the evidence regarding the effectiveness of CTs in alleviating the symptoms experienced in all three phases (pregnancy, delivery and the postpartum period) is still limited, pointing out that one of the possible reasons might be the low methodological quality of the clinical trials conducted.

Given the scarce evidence of studies jointly exploring the effectiveness of these therapies in all three maternal health phases, the objective of this systematic review is to analyze the benefits of complementary therapies during pregnancy, delivery and the postpartum period.

## 2. Materials and Methods

### 2.1. Search Strategy and Selection Criteria

From May to June 2024 an extensive search of potentially eligible trials was carried out by two independent reviewers (C.L.-D.-L.-I. and E.M.) in the following databases: Medline (Pubmed), Scopus, Web of Science and CINHAL (Comulative Index to Nursing and Allied Health Literature). PRISMA (Preferred Reporting Items for Systematic Reviews and Meta-Analysis) [29] guidelines were followed. To ensure no similar documents were overlooked, a search was conducted in the PROSPERO (Prospective Register of Systematic Reviews) registry and, additionally, Google Scholar was examined to reduce the risk of publication bias. The protocol of this review was registered in the PROSPERO database (CRD42024543981).

According to the Medical Subject Headings Thesaurus (MeSH)’s search terms, the search strategy used was: (“Complementary Therapies” AND (“Pregnancy” OR “Preg-nant women” OR “Postpartum Period” OR “Obstetric delivery” OR “Parturition” OR “Obstetric labor”)).

The “PICOS” model [30] was utilized to select the studies for this systematic review. The criteria for the selection were as follows: P (Population) = pregnant and postpartum women; I (Intervention) = complementary therapies; C (Comparison) = no comparison, no treatment, other techniques/conventional therapy; O (Outcomes) = benefits of complementary therapies during pregnancy, delivery and postpartum period; S (Type of study) = randomized controlled trial. Consequently, the research question was: Are complementary therapies beneficial during pregnancy, delivery and postpartum?

Only available articles in English, Spain and Italian related with the research question and without restriction of time were included.

Studies involving subjects under the age of 18 or with a PEDro scale’s score less than 6 were excluded.

### 2.2. Quality and Biask Risk Assesstment

The PEDro scale [31] was used to assess the methodological quality of the studies included in this systematic review. This scale examines the internal and external validity of studies based on 11 criteria: (1) specification of selection criteria; (2) random allocation; (3) concealed allocation; (4) similarity in baseline characteristics of groups; (5) blinding of subjects; (6) blinding of therapists; (7) blinding of assessors; (8) outcome measured for at least 85% of participants; (9) results reported for the entire sample, using “intention to treat” analysis for participants lost to follow-up; (10) statistical comparison between groups; (11) variability in outcome measurement [32]. Each criterion adds one point, and the final score is calculated without including the first criterion. Based on the score achieved, studies were considered to have excellent (9–10), good (6–8), fair (4–5), or poor (less than 4) methodological quality [33].

The risk of bias of the included clinical trials was evaluated using the revised Cochrane Risk of Bias tool for randomized trials, version 2 (RoB-2) [34,35]. Studies were assessed across five risk-of-bias domains: randomization process, deviations from intended interventions, missing outcome data, outcome measurement and selection of the reported result. Each type of risk was classified into three categories: low, some concerns, or high. The combined scores from each domain produced an overall score, ranking the study’s risk of bias as “Low”, “Some Concerns”, or “High” [34,35].

All assessments were carried out by two independent reviewers (C.L.-D.-L.-I. and E.M.), with a third reviewer (M.D.G-M.) intervening in case of disagreement.

### 2.3. Selection Process and Data Extraction

Two reviewers (C.L.-D.-L.-I. and E.M.) independently conducted the research and selection of articles, following a strict four-step procedure. In the first step, reviewers searched for and removed duplicate studies, identifying suitable studies by reading titles and abstracts. The second step involved excluding studies whose title or abstract did not align with the objective of the review. In the third step, full-text studies were thoroughly examined to ensure they met the inclusion criteria. Finally, the two reviewers independently analyzed the methodological quality of the selected studies using the PEDro scale, selecting only studies with a score higher than six, to guarantee a good methodological quality. Throughout the entire process, in cases where consensus on the selection of studies was not reached, a third reviewer (M.D.G-M.) was consulted.

A data coding manual was followed to extract information from the selected studies, which included: (1) author’s name, year of publication and country; (2) study objective; (3) study design and sample characteristics; (4) period of application (pregnancy, childbirth, or postpartum); (5) type and duration of intervention; (6) outcomes; and (7) main findings. Data extraction was also conducted independently by two researchers (C.L.-I. and E.M.), who systematically organized the information in a table using Word (Office Professional Plus 2016 for Windows, Microsoft, Redmond, WA, USA).

## 3. Results

**Figure 1:**
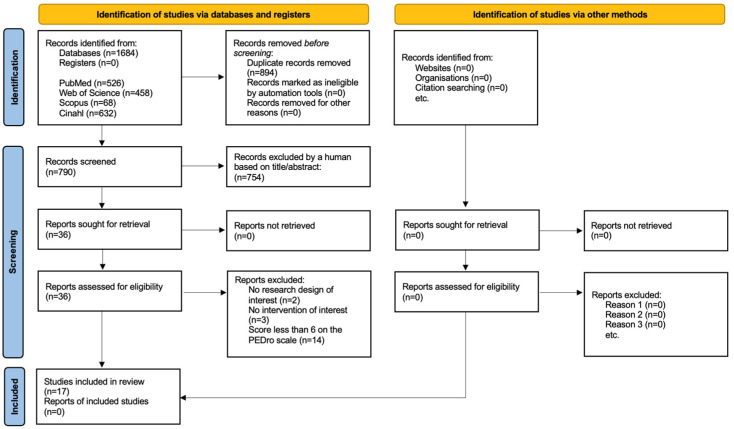
PRISMA 2020 flow chart for new systematic reviews, which included searches of databases, registers and other sources [29].

A total of 1684 studies were identified, with 17 being selected that met the inclusion criteria and had their methodological quality classified as good. Figure 1 shows the selection process. The PEDro scale scores obtained by the studies that were excluded are presented in the Supplementary Material of this article (Table S1).

### 3.1. Synthesis of the Results and Caracteristics of the Studies

The most important characteristics of the studies included [36,37,38,39,40,41,42,43,44,45,46,47,48,49,50,51,52] are presented in Table 1. Most of the studies (63%, n = 13) were published between 2018 and 2023 [37,39,40,42,43,44,45,47,48,49,50,51,52], with 2022 being the year with the highest number of publications (24%, n = 4) [43,49,50,52], followed by 2020 (18%, n = 3) [45,47,48] and by 2018 (18%, n = 3) [39,42,51].

More than half of the studies (n = 9, 53%) [38,39,40,44,45,48,49,50,52] were conducted in the Asian continent.

In relation to the phases during which they were carried out, most of the studies were developed for the postpartum period (41%, n = 7) [36,38,40,48,49,51,52] and the delivery phase (41%, n = 7: labor = 6; C-section = 1) [37,39,42,43,44,47,50], with pregnancy as the least studied phase (18%; n = 2) [41,45,46].

Among the studies, in two of them [36,46], the effect size of the obtained results was calculated, while in three others [44,50,51], the effect size was used a priori for the sample size calculation. In one study [52], both calculating the effect size and using it a priori for sample size were carried out.

### 3.2. Characteristics of the Participants

A total of 2024 subjects were included in this systematic review. All subjects were female: 359 were in the pregnancy phase [41,45,46], 814 were in the delivery phase [37,39,42,43,44,47,50] and 842 were in the postpartum phase [36,38,40,48,49,51,52]. The subjects’ most relevant sociodemographic characteristics are presented in Table 2.

### 3.3. Characteristics of the Interventions

The most commonly used CTs in the studies were aromatherapy (n = 4; 23.5%) [38,39,43,52], yoga (n = 3; 18%) [36,41,46], acupressure (n = 2; 12%) [43,45] and massage (n = 2; 18%) [50,51]. Most of the complementary therapies used (n = 10; 58.82%) are based on purely oriental techniques [36,40,42,45,46,50,52] or on their adaptation to the Western World [41,47,49].

Two studies [37,52] researched two complementary therapies simultaneously (hot shower baths and perinatal Swiss ball exercises in one of them [37] and aromatherapy and Su Jok in the other [52], respectively), verifying their benefits when used alone and in combination.

Regarding the phases in which the CTs were applied, only yoga [41,46] and acupressure [45] were used in the pregnancy phase; these two techniques were also employed during the postpartum phase in other studies [36,42]. Another technique that was used in more than one phase was aromatherapy, which was resorted to in the delivery [39,43] and postpartum [38,52] phases. Specific techniques such as hot shower baths and/or perinatal Swiss ball exercises [37] or virtual reality [44] were applied during labor. Specific techniques were used for C-sections, either pre-surgery (such as the binaural-based technique [47]) or post-surgery (such as electropuncture [40], phytotherapy [48], connective tissue massage [51] or Su Jok [52]). Specific interventions were also identified in the postpartum phase, such as mindfulness [49] or reflexology massage, which was used both during labor and in the postpartum period [50].

Regarding duration of the interventions, four studies involved longer interventions (8 weeks [36,46,49] or 2 intensive days [41]) whereas the rest [37,38,39,40,42,44,45,47,48,49,50,51,52] lasted less than an hour at the most, with 1 to 4 sessions.

Table 3 shows the CTs identified in the studies selected, as well as the phase (pregnancy, delivery, postpartum) when they were applied.

### 3.4. Main Outcomes

The principal outcome measured was the pain [37,38,40,42,44,48,50,51,52] follow by anxiety [36,37,44,46,47] and depression [36,41,46].

Pain was assessed using Visual Analog Scale (VAS) in eight studies [37,38,40,42,48,50,51,52], with seven of them reporting a significant reduction in pain associated with the following interventions: hot shower bath (*p* = 0.001) [37] and acupressure during labor (*p* < 0.0001) [42]; aromatherapy (*p* < 0.001) [38,52], electropuncture (*p* < 0.001) [40], connective tissue massage (*p* < 0.001) [51], individualized attention (*p* = 0.046) [51] and su jok (*p* < 0.000) [52] in postpartum Cesarean (*p* < 0.001) [40]; reflexology massage in postpartum (*p* < 0.001) [50]. In one study [44], the Numerical Pain Rating scale (NPRSN) was used to measure pain, showing a significant decrease in pain intensity (*p =* 0.004) with the use of virtual reality.

Anxiety and depression were assessed with difference scales, as we can see in Table 1. Four studies [36,44,46,47] reported a statistical diminution of anxiety when applying the following interventions: yoga during pregnancy (*p* < 0.001) [46] and in postpartum (*p* < 0.05) [36], virtual reality during labor (*p =* 0.003) [44] and a binaural-based technique or music before Cesarean (*p* < 0.0001) [47]. Depression significantly decreased in two studies [36,46], which both involved, respectively, an 8-week yoga program (*p* < 0.05; *p* < 0.04).

Nausea and vomit were investigated in only one study [45], finding a significant reduction (*p* < 0.001) in the Rodhes Index of Nausea, Vomiting and Retching’s score for nausea.

In relation to delivery, the use of medication was recorded in five studies [40,41,42,50,51] with a significant decrease in its use being found in four of them, which involved elctroacupuncture [40], a two-day specific intervention program for childbirth preparation [41], connective tissue massage [51] and reflexology massage [50]. Focusing on the labor, cervical dilatation was reported in two studies [37,43], where a significant improvement was linked to hot shower baths and/or perineal exercise with Swiss ball [37]. Among the studies that involved C-section, scar recovery was assessed using the Edema, Ecchymosis, Discharge, Approximation (REEDA) scale in one study (Pour) [48], where phytotherapy coincided with a significant improvement in Cesarean’s recovery.

Three studies [41,43,44] evaluated the neonate’s status using APAGAR score, with none finding significant difference associated with these interventions.

### 3.5. Assessment of Methodological Quality and Risk of Bias

All the studies included in this systematic review were RCTs: 30% (n = 5) were single-blind [36,38,40,41,50], 12% (n = 2) double-blind [45,47], 12% (n = 2) triple blind [43,48] and the remaining 47% (n = 8) [37,39,42,44,46,49,51,52] non-blind.

Regarding the methodological quality, 14 studies (82%) were considered to have a good methodological quality, with the following being achieved on the PEDro scale: for six studies [36,42,44,46,51,52], a score of six; seven studies [37,38,39,40,41,49,50] a score of seven; and one study [47] a score of eight. A total of three studies (18%) were rated as excellent, two of them achieving a PEDro score of 9 [43,45] and one a PEDro score of 10 [48]. The full scores achieved by each included study can be found in the Supplementary Material (Table S2).

**Figure 2:**
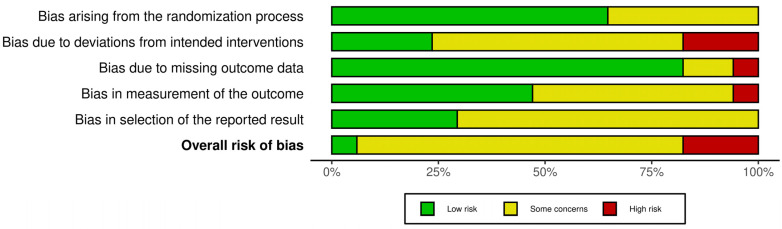
Bias assessment plot of included studies.

**Figure 3:**
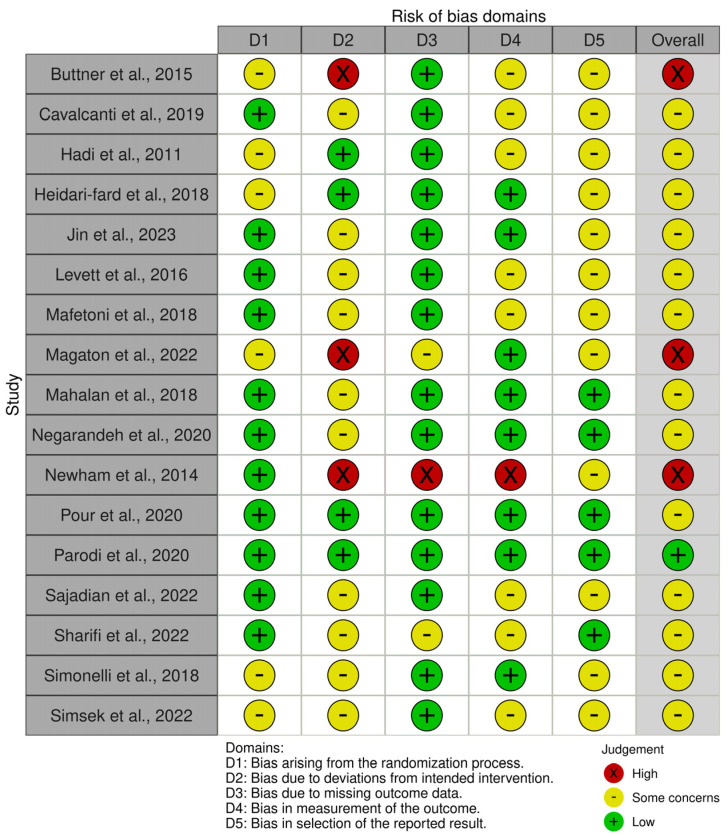
Bias assessment shown as a one-to-one summary plot [36,37,38,39,40,41,42,43,44,45,46,47,48,49,50,51,52].

About the risk of bias, as shown in Figure 2 and Figure 3, the largest number of studies (n = 13, 76%) had some concerns in the overall risk of bias judgment [37,38,39,40,41,42,43,44,45,48,49,50,51,52]. Only one study (6%) was judged to have an overall low risk of bias [47]. Bias due to deviations from intended interventions and biases in selection of the reported result were the most common. High risks of bias were present in the following bias’s domains: deviation from intended intervention, missing outcome data and in the measurement of the outcome.

## 4. Discussion

This systematic review analyses the benefits of CTs during pregnancy, delivery and the postpartum period. Only studies a minimum of good methodological quality were included, as assessed by means of the PEDro scale. Most of these studies were focused on researching the benefits of CTs in relation to pain management during delivery or in the postpartum period. Other variables of interest included anxiety, depression, medication use and newborns’ health. Among the CTs studied, a significant reduction in pain was observed during labor when using hydrotherapy, perinatal Swiss ball exercises, acupressure, virtual reality and foot reflexology. On the other hand, significant benefits were found for pain after C-sections when resorting to aromatherapy, electroacupuncture and massage. When practiced during pregnancy, yoga showed a significant reduction in anxiety and depression levels, a benefit that was also noticed when this practice was continued in the postpartum period. Interventions such as hot shower baths, perinatal Swiss ball exercises and virtual reality effectively reduced anxiety related to the delivery process. Finally, the binaural technique was the only one specifically studied to reduce the anxiety associated with C-sections, showing significant results in favor of its use.

### 4.1. Characteristics of the Participants and Studies

More than half of the studies were conducted in the Asian continent, with Iran as the country contributing the highest number of materials. This fact evidences how CTs based on oriental techniques [38,39,40,48,49,50,52] are more put into practice in nations where they more accessible, affordable and culturally accepted, being integrated into the health systems themselves [53,54].

It is worth noting that, in the studies conducted in Asian countries where information about marital status is provided [44,45,49,50], the entire samples are comprised by married individuals, whereas this marital status is only found in part of the sample in the studies from western countries [37,42,43,46,51]. Raymo et al. (2015) suggest that there is a strong cultural tradition of early marriage linked to procreation in eastern countries [55].

### 4.2. Characteristics of the Interventions

The results obtained about the prevalence of the CTs used in the studies included, as well as the phases during which they are applied, are in line with the existing literature. Aromatherapy turned out to be the most used [38,39,43,52], both during delivery and in the postpartum period, and was widely studied by other authors [16,23,25]. According to these studies, its use seems to be beneficial for pain management during labor [39,43] and in the postpartum period after C-sections [38,52]; however, it appears not to influence delivery progress [39] or cervical dilation [43].

Yoga has been researched both during pregnancy [41,46] and in the postpartum period [36], focusing on psychological benefits such as reduced anxiety and depression. The study conducted by Newham et al. (2014) corroborates that practicing yoga during pregnancy is associated with anxiety and depression reductions in the postpartum period [41]. The review by Corrigan et al. (2024) [27] supports these findings. However, the study by Levett et al. (2016) found no significant differences between the groups in relation to the onset of postpartum depression at 6 weeks [41], which might be attributed to the characteristics of the intervention: intensive (two days) and combined with other techniques in addition to yoga. In turn, the study by Newman et al. (2014) was exclusively focused on yoga [46] and lasted 8 weeks, as was the case with Buttner et al. (2015), which concluded that postnatal yoga also exerts a positive influence on reducing postpartum anxiety and depression, suggesting that its practice is beneficial in this phase as well [36].

Acupressure was employed to reduce nausea and vomiting during pregnancy [45] and for pain management during labor [42]. As for its use in reducing nausea and vomiting, the results found are inconclusive: in the study by Nagarandeh et al. (2020), acupressure seems to exert a positive effect on reducing nausea but not vomiting [45] whereas a recent review with meta-analysis [20] found that it was in fact capable of reducing vomiting, although with a low level of evidence. Regarding its efficacy in pain management during delivery, the evidence continues to be inconclusive [21,42].

Massage has been used to reduce pain both during labor [50] and in the postpartum period after C-sections [51]. Although both studies showed pain reductions and recorded benefits in the postpartum period, the two massage techniques employed were very different from each other: foot reflexology was used in one case [50] and connective tissue massage in the other [51]. However, both techniques seem to lack solid evidence in the literature. For example, the most recent review by Smith et al. (2018) about massage and hand techniques to reduce pain during delivery did not include any studies on foot reflexology [26]. Likewise, the benefits contributed by connective tissue massage [51] were analyzed along with other massage techniques in a specific review on complementary therapies during the postpartum period after C-sections [16], where no clear evidence about its effectiveness was found.

As for the phase when the CTs are applied, it is to be noted that, according to previous studies [27,56], interventions based on yoga and relaxation techniques [41,46] are the most commonly used during pregnancy, whereas a wide variety of techniques to address pain and psychological symptoms prevail during labor [21,26]. This heterogeneity among the techniques used in this phase is in line with the studies found in this review [37,39,42,43,44,50]. According to the existing literature [16,23], they are focused on a large variety of techniques to address pain in some cases [38,40,48,51,52] and for psychological symptoms in others [36,49] during the postpartum period as well.

In the studies where two CTs were applied simultaneously [37,52], neither of them turned out be more effective than the other. Thus, the benefits contributed by hot shower baths during delivery are comparable to those obtained with Swiss ball exercises [37], and the benefits of aromatherapy in the postpartum period are similar to those of Su Jok [52].

Finally, only four studies [36,41,46,49] included long interventions, all coinciding in the use of relaxation techniques (such as yoga or mindfulness). The interventions were shorter in the rest of the studies [37,38,39,40,42,43,44,45,47,48,50,51,52], with application times varying from 30 s [45] to 30 min [37,52]. Therefore, length of the interventions in time seems to be specific to each technique, which precludes making any comparisons regarding efficacy related to this aspect.

### 4.3. Main Outcomes

Pain was the most studied symptom in the studies included in this review, which is in line with the scientific literature [16,21,26]. In relation to pain during delivery, the techniques that seem to be effective are hot shower baths, Swiss ball exercises [37], acupressure [42] and foot reflexology massage [50]. An aspect shared by all these techniques is their application time of between 20 and 30 min, which renders them easy to apply in the clinical practice. Acupressure and foot reflexology massage (both oriental in origin) require previous training to be properly applied [42,50]. In turn, hot shower baths and Swiss ball exercises are easier to perform and their effectiveness is well documented for their use at the time of delivery [26,57].

Regarding how postpartum pain was approached, all the studies included in this review were conducted with women subjected to C-sections [38,40,48,51,52]. Phytotherapy was the only intervention in which no significant pain reduction was observed [48], possibly due to the fact that it was applied in the form of suppositories, using the same route of action of the analgesics to which it was compared. In the two studies that assessed aromatherapy [38,52], it seems that duration, inhalation frequency and type of oil used exerted no influence on the benefits obtained, because different protocols were used, with both turning out to be effective for pain management. In their study about CTs applied to women subjected to C-sections, Zimpel et al. (2020) [16] identified a new complementary therapy: electropuncture [40].

As for delivery-related anxiety, virtual reality [44] and the technique based on binaural sounds [47] proved to contribute significant benefits, a finding that is in line with those by Hunter et al. (2023), who support music therapy use [28]. These two techniques might be considered within this category, as they resort to music in their development. In addition, the benefits of the binaural technique can be compared to those contributed by music therapy, as observed in the study by Parodi et al. (2020), who compared them and obtained significant results in both [47]. The studies differ as for the delivery type addressed: virtual reality [44] was used during the active phase; in turn, the binaural technique [47] was applied before C-sections.

Anxiety was researched along with depression both during pregnancy [46] and in the postpartum period [36], where practicing yoga has been shown to contribute significant benefits in reducing these symptoms, as already described. According to Jorbour et al. (2022), practicing yoga during pregnancy might reduce postpartum depressive symptoms in pregnant women that have already presented previous depression episodes [56]. In line with these findings, the study by Newman et al. (2014) showed that the yoga intervention not only significantly reduced the depression levels but also fear of delivery [46], which might be a protective factor against developing postpartum depression [14].

As for nausea and vomiting, despite being a problem experienced by a high percentage of pregnant women [6], only one of the studies included in this review addressed these symptoms [45]. As already mentioned, acupressure seems to be a promising complementary therapy for treating them [20,45]; however, it is still necessary to develop higher levels of evidence supporting its use.

As for medication use, the studies found are heterogeneous in terms of pregnancy phase, intervention type and medication employed [40,41,42,51,52]. In general, both the techniques applied during pregnancy [41] and those performed at the time of delivery [50] or after a C-section [40,51] seem to exert a positive effect on reducing medication use during delivery. When applied during labor, acupressure [42] was the only technique that failed to show benefits, a finding also detected by Smith et al. (2020) [21]. Although the studies conducted after C-sections [40,51] recorded a significant reduction in medication use, Zimpel et al. (2020), failed to find any clear relationship between these two variables in their review [16]. Consequently, this is an interesting aspect to be explored in future research studies.

In relation to delivery progress (measured in terms of cervical dilation), when applied for 30 min during labor, techniques such as hydrotherapy and Swiss ball exercises [37] seem to contribute to this progression; in turn, other techniques (such as aromatherapy) appear not to exert the same effect [42]. This might be due to the fact that the former are interventions which directly involve women’s bodies, with a specific focus on the low back and pelvic areas [57], whereas aromatherapy exerts no direct physical action because it is applied through inhalation. These results are in line with the existing literature, which has found a relationship (albeit weak) between labor duration and using techniques such as massage, hot compresses and thermal–manual methods [26].

Finally, using complementary therapies during pregnancy and delivery seems not to offer significant benefits for newborns. According to previous studies [21,26], none of the three studies included in this review [41,43,44] found a positive relationship between using these therapies and newborns’ health.

### 4.4. Limitations

This review presents some limitations. In the first place, although on the one hand an attempt has been made to provide a global view of the relationships between them, the objective of analyzing the benefits contributed by the CTs, encompassing all maternal health phases, may have caused a certain amount of data dispersion, either in terms of selection of the studies or as for the interventions found. In fact, another limitation is the heterogeneity of the interventions, as it has hindered comparing them exhaustively. In addition, having applied methodological screening exerted a negative influence on the number of studies selected, which turned out to be insufficient to develop a stronger level of evidence. Among the selected studies, we identified methodological limitations that could be improved, such as bias caused by deviations from the intended intervention and the absence of effect size calculations in the majority of the studies. It is necessary to conduct a more detailed analysis of the results, including the calculation of the effect size and an intention-to-treat approach to account for follow-up losses. Finally, although a common pain measure (VAS) was identified, no meta-analysis of the studies was performed, which limited the strength of the findings. More studies with larger sample sizes and lower risks of bias need to be carried out to develop stronger evidence about the effectiveness of CTs in maternal health.

## 5. Conclusions

Complementary therapies contribute benefits in all maternal health phases. Hydrotherapy, perinatal Swiss ball exercises, acupressure, virtual reality and foot reflexology have proven to reduce pain during labor. Aromatherapy, electroacupuncture and massage have shown benefits in pain management after C-sections. When practiced during pregnancy, yoga can be a suitable intervention to reduce anxiety and depression, both during the prenatal and postnatal periods. Even though more clinical trials are required to confirm their effectiveness, they seem to be useful for supporting maternal health in clinical practice.

## Figures and Tables

**Table 1:** Most significant characteristics of the included studies.

Authors, Country (Year)	Design/Sample (Age ± SD, Years or Median with IQR)	Intervention/ Dosis	Main Outcomes and Measurement/Moment	Results
Buttner et al. (2015) [36] USA	RCT (S-B) n = 57 EG: 28 (29.81 ± 5.17) CG: 29 (32.45 ± 4.78)	EG: Yoga CG: Wait list control 8 wks: 1 h/1 time per wk	Anxiety: TI Depression: HDRS, GD, WB, SA Quality of life: SF-36 Pre-intervention, 2 wks, 4 wks, 6 wks, post-intervention	Significant reduction in depression (large effect size; HDRS and GD, *p* < 0.005; WB and SA, *p* < 0.05) and anxiety (TI, *p* < 0.05). Significant increase in quality of life (SF-36, *p* < 0.001)
Cavalcanti et al. (2019) [37] Brazil	RCT (O-L) n = 128 HBG: 44 (26.05 ± 5.41) SBG: 45 (27.24 ± 6.47) CTG: 39 (24.56 ± 4.91)	HBG: hot shower bath SBG: perinatal exercise with the Swiss ball CTG: hot shower bath and perinatal exercise with the Swiss ball 30 min during labor	Anxiety: VAS Pain: VAS Cervical dilatation: manual measurement Number of contractions in 20 min: cardiotocography Pre- and 30 min post-intervention	Reduction in anxiety across the entire sample, with no statistically significant difference between groups. Increase in pain in all three groups, with a statistically significant difference in the HBG (*p* = 0.001). Statistically significant difference in cervical dilation across all three groups (*p* < 0.001). Increase in the number of contractions in all three groups, with no statistically significant difference
Hadi et al. (2011) [38] Iran	RCT (S-B) n = 200 EG: n = 100 (24.72 ± 3.70) CG: n = 100 (25.02 ± 3.97)	EG: aromatherapy (lavender) CG: inhale a neutral aromatic material 3 min at 3, 8 and 16 h post-CS	Pain: VAS Pre-Cesarean, 8 and 16 h post-CS	Significant reduction in pain in EG (*p* < 0.001)
Heidari-fard et al. (2018) [39] Iran	RCT (O-L) n = 130 EG: n = 65 (25.58 ± 6.18) CG: n = 65 (26.86 ± 5.82)	EG: aromatherapy CG: inhale distilled water 3-times from 4 cm dilatation until the end of delivery	Contractions: Number, duration and intensity registered by examination and observation 3–4 cm, 5–7 cm and 8–10 cm of dilatation Satisfaction: 3-point Likert scale (satisfied, relatively satisfied and dissatisfied) Postpartum	No significant difference in the duration and number of contractions between the two groups. Significant reduction in contraction intensity at 5–7 cm in the EG (*p* < 0.004). Significant difference in satisfaction between the EG and CG (*p* < 0.0001), with higher satisfaction in the EG
Jin et al. (2023) [40] China	RCT (S-B) n = 174 EG: 53 (31.19 ± 3.37) EG_2_: 53 (30.13 ± 3.31) CG: 52 (31.19 ± 3.37)	EG: PCIA + 2 Hz electroacupuncture EG_2_: PCIA + 20/100 Hz electroacupuncture CG: PCIA + sham electroacupuncture 4/25 min sessions at 6, 12, 24 and 48 h post-CS	APC: number Fentanyl consumption during PCIA: number Pain: VAS Safety: number of adverse events 6, 12, 24 and 48 h post-Cesarean	Significantly higher number of APC in the CG (*p* < 0.001). No significant difference between the EG and EG2. Significant reduction in pain in both the EG and EG2 at 12, 24 and 48 h post-Cesarean (*p* < 0.001). Significant difference in fentanyl consumption in both the EG and EG2 at all time points (*p* < 0.05)
Levett et al. (2016) [41] Australia	RCT (S-B) n = 172 EG: 87 (28.87 ± 5.24) CG: 85 (30.41 ± 4.99)	EG: specific intervention program CG: usual care 2 days or 1 day per wk for 6–7 wks	Use of epidural: yes/no Augmentation of labor: yes/no Length of labor: minutes (first or second phase) Type of delivery: CS or NVB Labor Personal Control: LAS Depression: EPDS 72 h and 6 months post-labor Neonates’ status: APGAR score 72 h and 6 months post-labor	Significant reduction in labor augmentation and epidural use in the EG compared to the CG (*p* < 0.0001). Second phase of labor significantly shorter in the EG (*p* < 0.05) Higher number of NVB in the EG (*p* < 0.01) and fewer CS cases (*p* = 0.017). Significant difference in LAS score (*p* < 0.01) but not in EPDS score in the EG compared to the CG No significant difference in APGAR scores between the groups
Mafetoni et al. (2018) [42] Brazil	RCT (O-L) n = 156 EG: 52 (23.9 ± 5.8) TG: 52 (25.1 ± 7.0) CG: 52 (22.7 ± 5.3)	EG: acupressure TG: superficial pressure in SF36 point CG: usual care 20 min during the labor	Use of medication: yes/no Use of anesthesia: yes/no Labor Pain: VAS Pre-intervention and 20 and 60 min post-intervention	No statistical difference in the use of medication or anesthesia among the three groups. Significant reduction in pain 60 min post-intervention in the EG compared to the TG and CG groups (*p* < 0.0001)
Magaton et al. (2022) [43] Brazil	RCT (T-B) n = 164 (153 analyzed) EG: 78 (25.6 ± 6) CG: 75 (24.5 ± 5.1)	EG: aromatherapy CG: inhale mineral water 4 times/1 h	Cervical dilatation: manual exploration (cm) Type of delivery: CS or NVB Neonates’ status: APGAR score Labor and post-labor	No significant difference in cervical dilation between the two groups. Higher number of NVB in the EG compared to the CG (*p* = 0.038). No significant difference in APGAR scores between the groups
Mahalan et al. (2023) [44] India	RCT (O-L) n = 76 (74 analyzed) EG: 37 (23.08 ± 3.84) CG: 37 (23.49 ± 3.66)	EG: virtual reality CG: standard care 1 h/2 session of 20 min + 10 min break) in labor’s active phase	Anxiety: AASPWL Pain tolerance: BPI Pain intensity: NPRS Neonates’ status: APGAR score Pre-, 20 and 50 min post-intervention	Significant difference in anxiety between the EG and CG (*p* = 0.003) Significant reduction in pain intensity (*p* = 0.004) but not in pain tolerance in the EG compared to the CG. No significant difference in APGAR scores between the two groups
Negarandeh et al. (2020) [45] Iran	RCT (D-B) n = 128 EG: 64 (30.46 ± 5.07) CG: 64 (29.95 ± 5.23)	EG: acupressure CG: sham pressure point 3 days/3 times per day/30 s	Nausea and vomit: INVR Pre- and post-intervention	Significant decrease in nausea (*p* < 0.001) but not in vomiting in the EG compared to the CG
Newmhan et al. (2014) [46] United Kingdom	RCT (O-L) n = 59 (51 analyzed) EG: 22 (31 ± 5) CG: 29 (31 ± 7)	EG: yoga CG: usual care 8 wks Pre-CS	Anxiety: STAI-T, STAI-S Depression: EPDS Fear of childbirth: WDEQ Cortisol: saliva sample Pre- and post-intervention	Significant difference in STAI-S score and cortisol (*p* < 0.001) in the EG compared to the CG. Significant decrease in depression in the EG (medium effect size; *p* < 0.04). Significant reduction in the fear of childbirth in both the EG (*p* < 0.0001) and the CG (*p* = 0.04)
Parodi et al. (2020) [47] Italy	RCT (D-B) n = 60 EG: 20 (no data) MG: 20 (no data) CG: 20 (no data)	EG: binaural-based technique MG: normal music CG: no music Pre-CS	Anxiety: STRAI-Y Pre- and post-intervention	Significant reduction in anxiety in both the EG and MG (*p* < 0.0001) compared to the CG
Pour et al. (2020) [48] Iran	RCT (T-B) n = 86 EG: 43 (28.9 8 ± 5.72) CG: 43 (29.35 ± 4.92)	EG: phytotherapy CG: usual care 3 times/8–16 and 24 h after CS	Pain: VAS Pre- and-post intervention Discomfort: SDS Recovery of CS (scar): REEDA 8 and 24 h after CS	No statistically significant difference in pain between the groups Significantly less discomfort in the EG compared to the CG (*p* < 0.001). Significant difference in CS recovery (*p* = 0.047), favoring the EG
Sajadian et al. (2022) [49] Iran	RCT (O-L) n = 40 EG: 20 (22.4 ± 3.3) CG: 20 (23.8 ± 4.4)	EG: Mindfulness-based intervention CG: usual care 8 wks/1 times per wk	Adaptation to maternal role: MRAQ Pre-, post-intervention and 3-mounts post- intervention	Increase in MRAQ scores in both groups post-intervention and at follow-up, with no statistically significant differences between the groups
Sharifi et al. (2022) [50] Iran	RCT (S-B) n = 100 (80 analyzed) EG: 40 (29.18 ± 4.93) CG: 40 (29.10 ± 3.62)	EG: foot massage (4 min) + foot reflexology (2 min) CG: foot massage (4 min) + sham foot reflexology (2 min) 2 times/10 min in thethird phase of labor and 2 h postpartum	Pain: VAS Mefenamic acid use: by dose (mg) 1–2–3–4 h postpartum	Significant reduction in pain at 3 and 4 h postpartum in the EG compared to the CG (*p* < 0.001). Statistically less use of mefenamic acid in the EG compared to the CG (*p* = 0.02)
Simonelli et al. (2018) [51] USA	RCT (O-L) n = 165 EG: 55 (32.25 ± 5.04) AIG: 55 (33.25 ± 4.52) CG: 55 (31.96 ± 4.45)	EG: perinatal massage AIG: individualized attention consists of a talk with the staff about the birth experience CG: usual care 20 min	Pain: VAS Stress: VAS Relaxation: VAS Opioid and NSAID use: number Pre- and 60 min post-intervention	Significant reduction in pain and stress in the EG (*p* < 0.001; *p* < 0.001) and the AIG (*p* = 0.046; *p* = 0.014). Significantly higher relaxation (*p* < 0.001) and lower opioid use in the EG
Simsek et al. (2022) [52] Turkey	RCT (O-L) n = 120 (29.55 ± 4.85) EG: 30 (no data) EG_2_: 30 (no data) EG_3:_ 30 (no data) CG: 30 (no data)	EG: aromatherapy EG_2_: su jok EG_3_: aromatherapy + su jok CG: usual care 30 min	Pain: VAS Pre-, post- and 30 min post-intervention	Significant reduction in pain immediately post-intervention and 30 min post-intervention in the EG (moderate effect size; *p* < 0.001), EG2 (*p* < 0.001) and EG3 (*p* < 0.001), with no significant difference between the groups

AASPWL: anxiety assessment scale for pregnant women in labor; AIG: individualized attention group; APC: analgesic pump compressions; cm: centimeters; CG: control group; CTG: hot ball–Swiss ball group; CS: Cesarean section; EG: experimental group; EG_2_: second experimental group; h: hour; D-B: Double-Blind; EPDS: Edinburgh Postnatal Depression Scale; HBG: hot ball group; HDRS: Hamilton Depression Rating Scale; Hz: Hertz; INVR: Rodhes Index of Nausea, Vomiting and Retching; GD: IDAS General Depression scale; min: minutes; MRAQ: Maternal Role Adaptation Questionnaire; mg: milligram; NPRS: Numerical Pain Rating scale; NSAID: nonsteroidal anti-inflammatory drugs; NVB: normal vaginal birth; LAS: Labor Agentry Scale; WB: IDAS Well-Being Scale; SA: IDAS Social Anxiety; O-L: Open-Label; PCIA: patient-controlled intravenous analgesia; REEDA: Redness, Edema, Ecchymosis, Discharge, Approximation; s: second; S-B: single-blinded; SF36: 36-Item Short-Form Health Survey; SBG: Swiss ball group; SDS: Symptoms Distress Scale; STAI-S: State Trait Anxiety Inventory-State; STAI-T: State Trait Anxiety Inventory-Trait; STRAI-Y: State-Trait Anxiety Inventory; TI: traumatic intrusions scale; TG: touch group; T-B: Triple-Blind; USA: United states of America; VAS: Visual Analog Scale; wk: week; WDEQ: Wijma Delivery Expectancy Questionnaire; WB: IDAS Well-Being Scale.

**Table 2:** Most relevant description of the sociodemographic characteristics of the sample in each study.

Authors, (Year)	Sample (Recruitment or Analyzed)	Race	Occupation (Employed)	Marital Status	Parity (Number or μ ± SD)	Gestational Age (wks: Number or μ ± SD)	Mode of Delivery
Buttner et al. (2015) [36]	n = 57	51 white	37		28 P, 29 M	-	-
Cavalcanti et al. (2019) [37]	n = 128	71 Caucasian/13 black/5 Asian/48 brown-skinned	-	58 married or stable relation	0.71 ± 0.27	39.69 ± 1.06	128 NVB
Hadi et al. (2011) [38]	n = 200	-	33	-	EG:0.78 ± 0.22 CG:0.59 ± 0.10	-	200 CS
Heidari-fard et al. (2018) [39]	n = 130	-	91	-	130 P	EG: 39.30 ± 1.3 CG: 39.24 ± 1.23	3 CS; 127 NVB
Jin et al. (2023) [40]	n = 174	-	-	-	-	38.32 ± 1.43	174 CS
Levett et al. (2016) [41]	n = 172	102 Caucasian/21 Asian/80 mixed-race	-	-	172 P	24 to 34	172 NVB
Mafetoni et al. (2018) [42]	n = 156	57 white/17 black/2 Asian	-	148 married or stable relation	78 P, 78 M	37	156 NVB
Magaton et al. (2022) [43]	n = 163	41 white/15 black/7 yellow/90 brown	-	39 married/5 stable relation		37 to 42	163 NVB
Mahalan et al. (2023) [44]	n = 74	-	3	74 married	-	EG: 38.86 ± 38.74 CG: 38.74 ± 0.92	74 NVB
Negarandeh et al. (2020) [45]	n = 128	-	13	128 married	-	6 to 16	-
Newmhan et al. (2014) [46]	n = 59	-	49	57 married or stable relation		EG: 22 ± 4 CG: 21 ± 3	-
Parodi et al. (2020) [47]	n = 60	-	-	-	-	37	60 CS
Pour et al. (2020) [48]	n = 86	-	9	-	12 P, 74 M	39	86 CS
Sajadian et al. (2022) [49]	n = 40	-	8	40 married	40 P	-	27 NVB, 13 CS
Sharifi et al. (2022) [50]	n = 80	-	2	80 married	-	37 to 42	80 NVB
Simonelli et al. (2018) [51]	n = 165	117 white/12 black/15 Asian/13 other/7 unknow/1 Native American	-	121 married/3 divorced	165 P	-	165 CS
Simsek et al. (2022) [52]	n= 120	-	100	-	20 P, 100 M	-	120 CS

CG: control group; CS: Cesarean section; EG: experimental group; M: multiparous; NVB: normal vaginal birth; P: primiparous; wks: weeks.

**Table 3:** Description of the interventions carried out in each study.

Authors, (Year)	Phase	Complementary Therapy
Buttner et al. (2015) [36]	Postpartum	Yoga: sun salutations, balancing, twisting and relaxation postures.
Cavalcanti et al. (2019) [37]	Delivery (labor)	Hot shower bath: water at 37° (measured with a digital Akso^®^ brand thermometer) directed as a jet onto the lumbar–sacral region, applied in a standing or seated position. Perinatal exercise with Swiss ball: propulsion and pelvic rotation movements in a seated position (legs flexed at 90°) using a 60 cm Gynboll^®^. Combined therapy: perinatal exercise with Swiss Ball performed during a hot shower bath.
Hadi et al. (2011) [38]	Postpartum (Cesarean)	Aromatherapy: lavender essence applied on a cotton swab and inhaled through an oxygen mask (2 drops of 2% Lavandula, from the mint family *Lamiaceae*, prepared by the pharmaceutical staff at Tabriz University of Medical Sciences).
Heidari-fard et al. (2018) [39]	Delivery (labor)	Aromatherapy: chamomile essence applied to a cotton swab and inhaled from a distance of 7–10 cm from the nose (2 drops of distilled Shiraz chamomile produced by Zarband Firm).
Jin et al. (2023) [40]	Postpartum (Cesarean)	Electroacupuncture: acupuncture performed at bilateral Zusanli (ST36) and Sanyinjiao (SP6) points using an Electric Acupuncture Stimulator (SDZIIB; Suzhou Medical Supplies Factory Co., Ltd., Suzhou, Jiangsu, China) with Jiachen Brand sterile disposable acupuncture needles (0.30 × 40 mm; Wujiang Jiachen Acupuncture Devices Co., Ltd., Suzhou, Jiangsu, China). A continuous 2 Hertz frequency or alternating 20/100 Hertz wave was administered with an intensity of 0.1–5.0 microamperes.
Levett et al. (2016) [41]	Pregnancy	Two-Day Specific Intervention Program: guided visualization, yoga postures, breathing techniques, massage, acupressure and facilitated partner support, utilizing the concept of “working with pain”.
Mafetoni et al. (2018) [42]	Delivery (labor)	Acupressure: deep pressure (±5 kg) applied to the Sanyinjiao (SP6) point with rapid decompression applied on the thumbs.
Magaton et al. (2022) [43]	Delivery (labor)	Aromatherapy: inhaled Five-Flower Formula™ imported by Healing Essências Florais^®^ (São Paulo, Brazil) (4 drops diluted in 20 mL of water).
Mahalan et al. (2023) [44]	Delivery (labor)	Virtual reality: slideshow of images depicting pregnant women or breastfeeding mothers, accompanied by Raga Desi Todi music. Device used: Samsung Galaxy J7 Prime smartphone with Procus ONE VR headset (40 mm lenses).
Negarandeh et al. (2020) [45]	Pregnancy	Acupressure: magnetic seed applied to Cardia, Point Zero, Shen Men, Sympathetic Autonomic and Stomach points, which the patient presses three times a day (morning, noon and night) for 30 s. Compressive pressure ranges from moderate to strong, applied up to the point of discomfort based on individual tolerance
Newmhan et al. (2014) [46]	Pregnancy	Yoga: postures and relaxation/breathing techniques, with postures and exercises based on antenatal yoga (a gentle adaptation of Hatha yoga). The program includes the following: basic yoga for common pregnancy ailments, postures for optimal fetal positioning, yoga techniques for the different phases of labor and practices for the postnatal period
Parodi et al. (2020) [47]	Delivery (Cesarean)	Binaural-Based Technique: Dynamic Multi-Spectrum Phase Shift (algorithm developed by EffettoVIOLA Medical), composed of infinite frequencies within bands based on specific brainwave patterns taken from deep states of yoga and meditative relaxation
Pour et al. (2020) [48]	Postpartum (Cesarean)	Phytotherapy: rectal administration of E. laciniata suppository
Sajadian et al. (2022) [49]	Postpartum	Mindfulness-Based Intervention (adapted from Vieten’s Mindful Motherhood Program). The intervention includes: conscious breathing, “being with baby” meditation, sitting meditation, walking meditation, body scan, loving–kindness meditation, mindful eating with a raisin, mindfulness exercises for daily life, self-acceptance and discussions about emotions and fears (adapted from Vieten’s Mindful Motherhood Program).
Sharifi et al. (2022) [50]	Delivery (labor)	Reflexology Massage: Reflexology foot massage applying rotational pressure to specific points associated with the pituitary gland, solar plexus and uterus.
Simonelli et al. (2018) [51]	Postpartum (Cesarean)	Connective tissue massage: perinatal connective tissue massage
Simsek et al. (2022) [52]	Postpartum (Cesarean)	Aromatherapy: Inhalation of lavender, eucalyptus, or rose essential oil (3 drops) applied to a gauze. Su Jok: technique using viable buckwheat seeds applied with a bandage on both hands in specific acupuncture points previously massaged

cm: centimeter.

## Data Availability

The protocol of this review was registered in PROSPERO database (CRD42024543981). Available from: https://www.crd.york.ac.uk/prospero/display_record.php?ID=CRD42024543981 (accessed on 27 November 2024).

## Supplementary Materials

The following supporting information can be downloaded at https://www.mdpi.com/article/10.3390/healthcare12232481/s1: Table S1: PEDro score of each item with the total score of the excluded studies. Table S2: PEDro score of each item with the total score of the included studies.
