# Supplementary material for: Benefits of Complementary Therapies During Pregnancy, Childbirth and Postpartum Period: A Systematic Review
Source: Healthcare (Basel). 2024 Dec 9;12(23):2481. doi: 10.3390/healthcare12232481 (PMC11640780; doi:10.3390/healthcare12232481)
Supplement: Supplementary file 1 [file healthcare-12-02481-s001.zip › healthcare-3332587-supplementary.pdf]

Table S1. PEDro score of each item with the total score of the excluded studies

Author/s and year	PEDro											
	1	2	3	4	5	6	7	8	9	10	11	T
Akmese et al., 2014	Y	Y	N	Y	N	Y	N	Y	N	Y	Y	5
Chia-Yen et al., 2011	Y	Y	N	Y	N	N	N	Y	N	Y	Y	5
Cicek et al., 2017	Y	N	N	Y	N	N	N	N	N	Y	Y	3
Dastjerd et al., 2023	Y	Y	N	Y	N	N	N	Y	N	Y	Y	5
Dehcheshmeh et al., 2015	Y	Y	Y	Y	N	N	N	N	N	Y	Y	5
Delgado-García et al., 2012	Y	Y	N	Y	N	N	N	Y	N	Y	Y	5
Klabbers GA. Et al., 2017	Y	Y	N	Y	N	N	N	N	Y	Y	Y	5
Yi-Li Ko et al., 2014	Y	Y	N	Y	N	N	N	Y	N	Y	Y	5
Mafetoni et al., 2016	Y	Y	N	Y	N	N	N	Y	N	Y	N	4
Mafetoni et al., 2015	Y	Y	N	Y	N	N	N	N	N	Y	Y	4
Nakamura et al., 2016	Y	Y	N	Y	N	N	N	Y	N	Y	Y	5
Sundar, et al., 2015	Y	Y	N	Y	N	N	N	Y	N	Y	Y	5
Taavoni et al., 2016	Y	Y	N	Y	N	N	N	N	N	Y	Y	4

Abbreviations: Y=yes; N=no; T=total

Table S2. PEDro score of each item with the total score of the inclueded studies

Author/s and year	PEDro											
	1	2	3	4	5	6	7	8	9	10	11	T
Buttner et al., 2015	Y	Y	N	Y	N	N	Y	N	Y	Y	Y	6
Cavalcanti et al., 2019	Y	Y	Y	Y	N	N	N	Y	Y	Y	Y	7
Hadi et al., 2011	Y	Y	N	Y	Y	N	N	Y	Y	Y	Y	7
Heidari-fard et al., 2018	Y	Y	Y	Y	N	N	N	Y	Y	Y	Y	7
Jin et al., 2022	N	Y	Y	N	N	N	Y	Y	Y	Y	Y	7
Levett et al., 2016	Y	Y	Y	N	N	N	Y	Y	Y	Y	Y	7
Mafetoni et al., 2018	Y	Y	Y	Y	N	N	N	Y	N	Y	Y	6
Magaton et al., 2022	Y	Y	Y	Y	Y	Y	Y	Y	N	Y	Y	9
Mahalan et al., 2023	Y	Y	Y	Y	N	N	N	Y	N	Y	Y	6
Negarandeh et al., 2020	Y	Y	Y	Y	Y	N	Y	Y	Y	Y	Y	9
Newham et al., 2014	N	Y	Y	Y	N	N	N	Y	N	Y	Y	6
Parodi et al., 2020	Y	Y	Y	Y	Y	N	Y	Y	N	Y	Y	8
Pour et al., 2020	Y	Y	Y	Y	Y	Y	Y	Y	Y	Y	Y	10
Sajadian et al., 2022	Y	Y	Y	Y	N	N	N	Y	Y	Y	Y	7
Sharifi et al., 2022	Y	Y	Y	Y	Y	N	N	Y	N	Y	Y	7
Simonelli et al., 2018	Y	Y	N	Y	N	N	N	Y	Y	Y	Y	6
Simsek et al., 2022	Y	Y	N	Y	N	N	N	Y	Y	Y	Y	6

Abbreviations: Y=yes; N=no; T=total
